# XGDAG: explainable gene–disease associations via graph neural networks

**DOI:** 10.1093/bioinformatics/btad482

**Published:** 2023-08-02

**Authors:** Andrea Mastropietro, Gianluca De Carlo, Aris Anagnostopoulos

**Affiliations:** Department of Computer, Control and Management Engineering “Antonio Ruberti”, Sapienza University of Rome, Rome 00185, Italy; Department of Computer, Control and Management Engineering “Antonio Ruberti”, Sapienza University of Rome, Rome 00185, Italy; Department of Computer, Control and Management Engineering “Antonio Ruberti”, Sapienza University of Rome, Rome 00185, Italy

## Abstract

**Motivation:**

Disease gene prioritization consists in identifying genes that are likely to be involved in the mechanisms of a given disease, providing a ranking of such genes. Recently, the research community has used computational methods to uncover unknown gene–disease associations; these methods range from combinatorial to machine learning-based approaches. In particular, during the last years, approaches based on deep learning have provided superior results compared to more traditional ones. Yet, the problem with these is their inherent black-box structure, which prevents interpretability.

**Results:**

We propose a new methodology for disease gene discovery, which leverages graph-structured data using graph neural networks (GNNs) along with an explainability phase for determining the ranking of candidate genes and understanding the model’s output. Our approach is based on a positive–unlabeled learning strategy, which outperforms existing gene discovery methods by exploiting GNNs in a non-black-box fashion. Our methodology is effective even in scenarios where a large number of associated genes need to be retrieved, in which gene prioritization methods often tend to lose their reliability.

**Availability and implementation:**

The source code of XGDAG is available on GitHub at: https://github.com/GiDeCarlo/XGDAG. The data underlying this article are available at: https://www.disgenet.org/, https://thebiogrid.org/, https://doi.org/10.1371/journal.pcbi.1004120.s003, and https://doi.org/10.1371/journal.pcbi.1004120.s004.

## 1 Introduction

Gene–disease association (GDA) discovery is one of the main tasks in network medicine. The goal of computational methods in this field is to prioritize what genes are more likely to be associated with diseases. This is usually performed by leveraging network data, such as protein–protein interaction (PPI) networks and gene–disease networks. Among the most used PPIs, we find, for instance, BioGRID (Oughtred *et al.* 2019), HuRI (Luck *et al.* 2020), and STRING (Szklarczyk *et al.* 2021). In these networks, nodes are proteins (or genes) that are connected with each other if an interaction exists. For gene discovery purposes, these networks are extended with information on disease associations, for which databases such as DisGeNET (Piñero *et al.* 2017, 2020) and eDGAR (Babbi *et al.* 2017) are typically used.

Many gene detection techniques have been developed over the years. Among the most known approaches are DIAMOnD (Ghiassian *et al.* 2015) and DiaBLE (Petti *et al.* 2020), which rely on the concept of *connectivity significance* for finding new candidate disease genes. Other techniques, such as ProDiGe (Mordelet and Vert 2011) and DOMINO (Quinodoz *et al.* 2017), use machine learning to determine associated genes. Another approach, Markov clustering (MCL) (Enright *et al.* 2002, Sun *et al.* 2011), creates clusters by applying *stochastic flow simulation* in graphs, and genes in the same clusters of associated genes are considered candidates. Another line of work uses random walks with restart (RWR) (Köhler *et al.* 2008, Valdeolivas *et al.* 2019) for the task of gene discovery. GUILD (Guney and Oliva 2012) leverages the paths interconnecting nodes corresponding to disease genes to derive topology-based rankings. ToppGene (Chen *et al.* 2009) makes use of a fuzzy similarity measure to compute the similarity between pairs of genes based on semantic annotations. Furthermore, gene discovery can be framed as a *positive–unlabeled* (PU) learning problem (Bekker and Davis 2020).

Differently from classic machine learning scenarios, in which a binary dataset consists of positive and negative samples, in PU learning instead of negative samples, we have a set of *unlabeled instances*, which can be regarded as a set of negative elements and some positive samples that have not yet been discovered. Different strategies approach gene discovery as a PU learning task by employing two-step techniques, such as PUDI (Yang *et al.* 2012), EPU (Yang *et al.* 2014), and, more recently, NIAPU (Stolfi *et al.* 2023).

Motivated by these previous studies, we frame gene prioritization as a PU learning problem. Given its performance, we rely on the NIAPU pipeline to define the node features and the label propagation system. Then, after the application of NIAPU, we train a GraphSAGE (Hamilton *et al.* 2017) model over the propagated labels. Finally, the explainability phase defines the explanation subgraph for associated genes that we use to expand the set of candidate genes for further analysis: we make the hypothesis that such genes may have newly associated genes, following the connectivity significance principle (Ghiassian *et al.* 2015), according to which a seed gene is likely to be connected to other seed genes. At first, we explore different *explainable artificial intelligence* (XAI) methods to determine the top-performing ones, and then we compare those selected with several state-of-the-art methods for disease gene identification. We call our proposed method XGDAG (eXplainable Gene–Disease Associations via Graph neural networks).

To the best of our knowledge, XGDAG is the first method to use an XAI-based solution in the context of PU learning for disease gene prioritization with graph neural networks (GNNs). The main contribution of the work lies in the novel use of the explainability results. Commonly, XAI is used as a passive tool to support and rationalize model decisions. In our case, explainability tools have an active role in the computation of the final ranking, given that the new candidate genes are directly extracted from the explanation subgraphs (see Section 3.3). This approach drastically diverges from previous attempts to use XAI for GNNs for a similar task. Indeed, Pfeifer *et al.* (2022) proposed the use of XAI to weight patient-specific PPIs before applying clustering for disease module detection. Even in this case, the use of XAI can be regarded as a support tool to enhance the output of other methods rather than an active tool to produce the final results.

## 2 Data sources and processing

We selected BioGRID (version: 4.4.206) as the PPI network for our experiments. We collected GDAs from DisGeNET (Piñero *et al.* 2015, 2017, 2020) (version: 7.0), considering 10 diseases: malignant neoplasm of breast (disease ID C0006142), schizophrenia (C0036341), liver cirrhosis (C0023893), colorectal carcinoma (C0009402), malignant neoplasm of prostate (C0376358), bipolar disorder (C0005586), intellectual disability (C3714756), drug-induced liver disease (C0860207), depressive disorder (C0011581), and chronic alcoholic intoxication (C0001973). Disease selection and data cleaning criteria are the same as in Stolfi *et al.* (2023). In particular, we considered diseases with a high number of seed genes, to allow for coherent learning of the neural network. We filtered the PPI to save interactions only between *Homo sapiens* genes. After isolating the largest connected component of the network, we ended up having a PPI consisting of 19 761 genes and 678 932 undirected links. Regarding GDAs, we removed genes that were not in BioGRID, resulting in 1025 genes for disease C0006142, 832 for C0036341, 747 for C0023893, 672 for C0009402, 606 for C0376358, 451 for C0005586, 431 for C3714756, 320 for C0860207, 279 for C0011581, and 255 for C0001973. To train our deep learning model, we considered GDAs from the *curated* set of associations, which contains GDAs from reliable sources (UniProt Consortium 2015, Davis *et al.* 2019, Rehm *et al.* 2015, Martin *et al.* 2019, Tamborero *et al.* 2018, Gutiérrez-Sacristán *et al.* 2015). Instead, as we describe in Section 4, for the validation of our methodology, we rely on the set of *all associations*. This is an extension of the dataset composed of GDAs gathered from additional sources not considered in the curated set (Bundschus *et al.* 2008, 2010, Bravo *et al.* 2014, 2015), and forms a solid base to evaluate the discovery efficacy of computational methods. An in-depth structural analysis of network properties is available in the Supplementary Material.

## 3 Methodology

**Figure 1.**
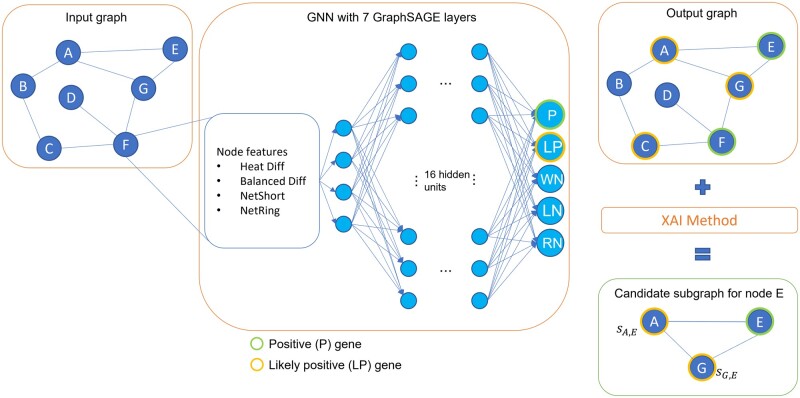
The XGDAG framework. A graph based on a PPI network and enriched with GDA information and node features is fed into a GNN. After the network has been trained, the predictions for the positive (P) genes are explained using an XAI methodology. Next, the nodes that appear in both the explanation subgraph and in the LP set are marked as candidate genes for prioritization.

We frame gene discovery as a PU learning problem. Our method is a three-step procedure that consists of (i) applying the NIAPU label propagation methodology to assign pseudo-labels to enable proper PU learning, (ii) training a GNN GraphSAGE model, and (iii) using explainability strategies for GNNs to compute explanation subgraphs for gene prioritization and define new putative disease genes. We now explain these steps, depicted in Fig. 1.

### 3.1 Label propagation

Our dataset can be seen as a PU dataset, in which a gene can be associated with a disease (*positive*) or not (*unlabeled*). Because associations may exist but not been discovered yet, it is not safe to mark unknown associations as *negative*. Moreover, PU datasets are usually highly unbalanced. In fact, only a small fraction of the entire set of genes in the interactome are associated with a given disease. Training on unbalanced datasets can negatively impinge on the performance of machine and deep learning models, and this results in the need for specific methods for unbalanced learning (Wang *et al.* 2021). For these reasons, label propagation procedures can be used to assign pseudo-labels to unlabeled instances, with a 2-fold benefit: avoid the bias introduced by setting the unlabeled instances as negative and obtain a more balanced dataset.

NIAPU (Stolfi *et al.* 2023) uses a Markovian diffusion process to assign four pseudo-labels to unlabeled genes according to the likelihood of association: *likely positive* (LP), *weakly negative* (WN), *likely negative* (LN), and *reliably negative* (RN). To do that, it relies on disease-specific features that allow the proper identification of the different classes (the positive class P and the pseudo-classes). In particular, it assigns to each gene, for each disease, the following features: heat diffusion (Carlin *et al.* 2017), balanced diffusion, NetShort (White and Smyth 2003), and NetRing (Baronchelli and Loreto 2006). Differently from classic network measures (degree, betweenness centrality, etc.), which only depend on the graph topology and are the same regardless of the disease considered, these features are computed taking into account the seed genes (represented by the class P). For this reason, for each disease, we have a different set of features assigned to the genes which properly characterize the disease itself. The NIAPU label assignment pipeline is composed of six core steps. In the first step, a gene similarity matrix is built, relying on the aforementioned features. As a second step, the similarity matrix is simplified by removing edges with weak connections, excluding them from the label propagation process. Third, the starting probabilities for the Markovian diffusion process are initialized and the RN set is defined to be the set of genes that are furthest from the genes in P. The fourth step is the Markov diffusion process itself, which distributes label probabilities across the graph. In the fifth step, the stationary distribution of the Markov process is used to assign the rest of the pseudo-labels. The sixth and last step consists in training a machine learning model (a GNN, in our case) on the newly assigned labels. More details on the features used, their effectiveness in gene discovery, and the NIAPU algorithm can be found in the work of Stolfi *et al.* (2023) and in the Supplementary Material.

### 3.2 Graph neural network model and training

After the label propagation, we obtain a dataset in which previously unlabeled items are labeled with the most suitable pseudo-label. We next train a GraphSAGE (Hamilton *et al.* 2017) GNN model. This is an inductive learning procedure that learns the embedding of a node assuming that the nodes in the same neighborhood have similar features. It does that by learning aggregator functions that generate node embeddings relying upon a node’s features and neighbors. A GraphSAGE layer, as defined in the PyTorch Geometric (Fey and Lenssen 2019) implementation we used, that generates the embedding $x_{i}^{\prime }$ for node *i*, after the application of a nonlinear activation function *σ*, has the following formula: where $W_{1}$ and $W_{2}$ are the weights learned by the neural network, $x_{i}$ is the feature vector for node *i*, $N_{\left(i\right)}$ is the one-hop neighborhood of node *i*, and $x_{j}$ is the feature vector for the neighbor node *j*. The mean function aggregates information from all the neighboring nodes without applying any sampling. In our case, *σ* is a ReLU function (Fukushima 1975). The use of this GNN is also suitable for dynamic graphs, as it is able to generate embeddings of new nodes without the need to retrain the model; only node features and neighbor node information is needed. Because a single layer aggregates information at a distance of one-hop and the diameter of our network is seven, we employ a seven-layer GraphSAGE GNN to gather the information flowing through the whole network. Working with deep GNNs may cause oversmoothing (Zhao and Akoglu 2020), which consists in the degradation of the model’s performance as the number of layers increases. To guarantee that this does not occur in our case, we tested different architectures with different depths, obtaining the best performance with seven GraphSAGE layers (the results of the competitive study are available in the Supplementary Material). We trained the model using the Adam optimizer (Kingma and Ba 2015) with learning rate set to 1e−3 and weight decay to 5e−4 for a maximum of 40 000 epochs, employing an early stopping procedure when the loss reaches a plateau. To train the model, we split the dataset into training (70%), validation (15%), and test sets (15%), maintaining the balance of the classes between the sets. The performances of the GNN on the test set are summarized in Table 1.


(1) $$x_{i}^{\prime }=\sigma \left(W_{1}x_{i}+W_{2}\cdot {\text{mean}}_{j\in N_{\left(i\right)}}x_{j}\right),$$

**Table 1.** Average results with standard deviation over the ten diseases for the GNN model.

Label	Precision	Recall	F1 score
P	0.956 ± 0.033	0.962 ± 0.064	0.958 ± 0.04
LP	0.876 ± 0.082	0.911 ± 0.077	0.888 ± 0.046
WN	0.861 ± 0.068	0.815 ± 0.11	0.831 ± 0.059
LN	0.868 ± 0.046	0.835 ± 0.066	0.85 ± 0.044
RN	0.858 ± 0.055	0.886 ± 0.06	0.871 ± 0.047
Macro avg	0.884 ± 0.027	0.882 ± 0.026	0.879 ± 0.028
Weighted avg	0.869 ± 0.031	0.863 ± 0.034	0.862 ± 0.035
Accuracy	0.863 ± 0.034		

### 3.3 Explainability phase

**Figure 2.**
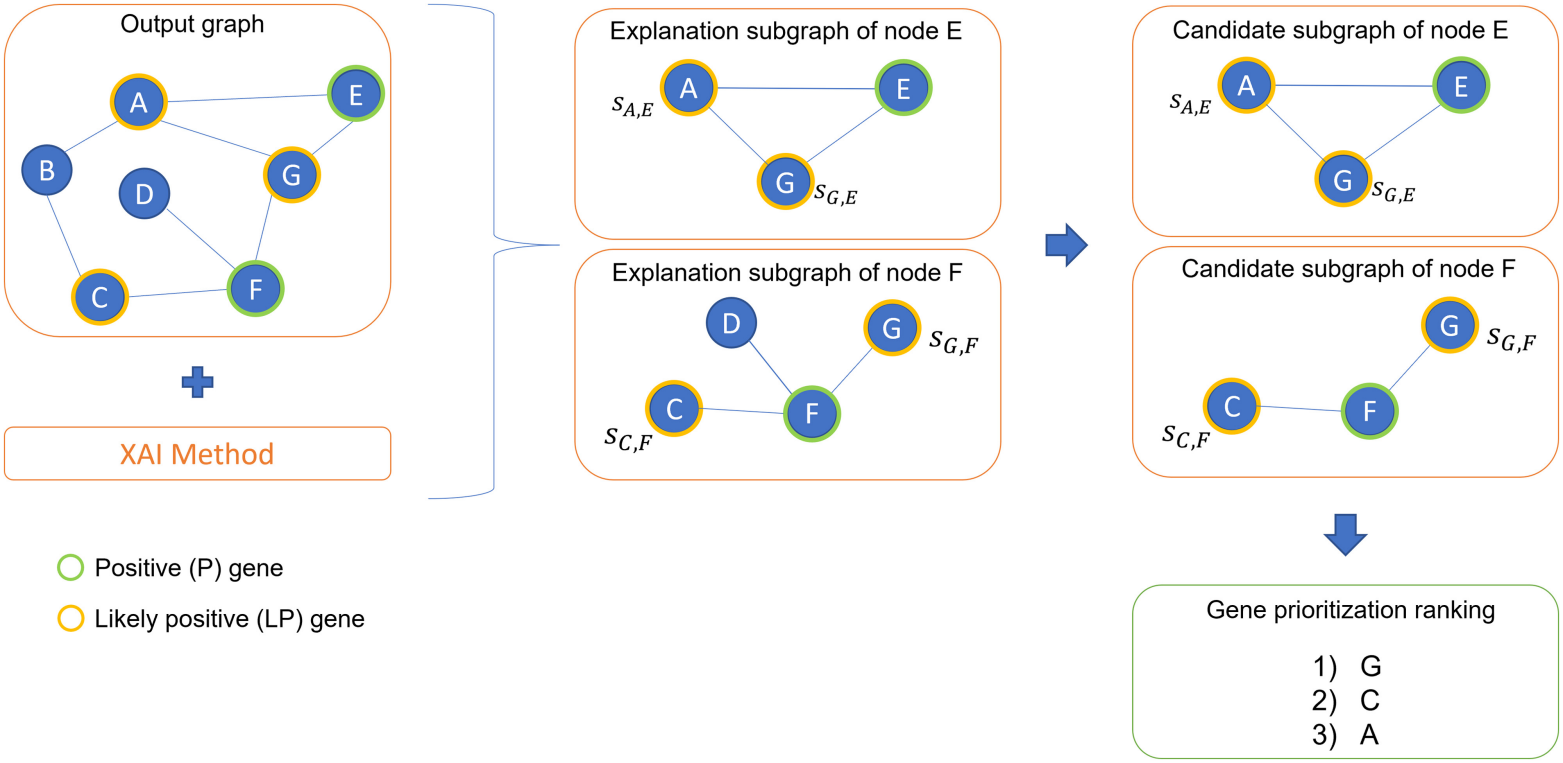
Graphical representations of the XGDAG prioritization mechanism. The output graph from the GNN is fed into an XAI method. For each P gene, we generate an explanation subgraph. This contains the nodes that were influential for the prediction of the node as P. We pool the subgraph by filtering out non-LP nodes, obtaining a final candidate subgraph. *s_ij_* is the importance score assigned by a given explanation method to *i* for the prediction of node *j*. Assuming the cumulative importance score for node C to be greater than the one of node A (*S_C_* > *S_A_*), we obtain the gene raking in the picture, with G as the top-ranked node because it appears in two candidate subgraphs.

The next step, after the training of the model, is to explain its predictions. For that, we have tested several XAI techniques on top of XGDAG. These methods output a subgraph of the original graph, the *explanation subgraph*, which contains the most influential nodes for the prediction. Our method applies one explainability technique to the positive genes P. For each explained node *n*, we thus obtain the explanation subgraph *G_n_*. Every node in *G_n_* has an importance score assigned (which depends on the XAI method used). *G_n_* may contain nodes belonging to different pseudo-classes. To enhance the accuracy of the results, we filter *G_n_* by keeping only the genes that the GNN predicted to be LP, which are more likely to be associated genes according to the NIAPU labeling. We thus obtain a reduced explanation subgraph, the candidate subgraph $G_{n}^{LP}$. We repeat this process for every node in P. If a node *i* appears in more candidate subgraphs, it is more likely to be associated with the disease, as per the connectivity significance property (Ghiassian *et al.* 2015). We take this into account as follows: we keep track of the number *M_i_* of subgraphs in which node *i* appears and of its cumulative importance score *S_i_*, obtained by summing all the importance scores *s_ij_* that node *i* has in the prediction of each node *j*—we assume that *s_ij_* = 0 if *i* is not in *G_j_*. Every gene *i* is then assigned a tuple (*M_i_*, *S_i_*). Finally, we obtain a ranking of candidate genes by sorting all the genes in the explanation subgraphs according to (*M_i_*, *S_i_*). A graphical representation of the XGDAG prioritization mechanism is shown in Fig. 2.

*Explainability methods for GNNs.* In our study, we made use of three XAI methods for GNNs. Each one of them relies on a different rationale to obtain explanation subgraphs. The first method is GNNExplainer (Ying *et al.* 2019), which established itself as the first explanation methodology for GNNs and it is still among the most used strategies for explaining GNN predictions. It works by learning a mask on the adjacency matrix by maximizing mutual information. Its output is a subgraph of nodes that are relevant for the prediction (along with a subset of node features). Its predictions are edge-oriented. Another method we used is GraphSVX (Duval and Malliaros 2021). It relies on a linear approximation of the concept of Shapley values (Shapley 1953) from game theory, which here are used as a proxy for node importance contribution. The use of Shapley values puts GraphSVX explanations on a solid and robust theoretical background. It delivers node-centric explanations. Finally, the third strategy is called SubgraphX (Yuan *et al.* 2021). It is the first methods to be focused on the research of explanation subgraphs only in terms of connected graphs, evaluating the importance that each of them has on the prediction. It exploits a Monte Carlo tree search to look for promising coalitions of connected nodes and computes a Shapley value approximation for each subgraph. The selected one is the subgraph associated to the highest Shapley value. The three methods explain the predictions leveraging the three different key components of a graph; edges, nodes, and subgraphs, respectively. This allows us to have comprehensive explanations of the GNN predictions.

To use XAI methods as independent tools for prioritization, we employ them in a PU learning setting. Indeed, we use them to explain models trained on binary PU data, devoid of any prior label propagation. As a result, they lack the assistance provided by the classes generated during the label propagation phase, which can be considered as a preliminary prioritization. Without the assistance of the LP class, the entire explanation subgraph is considered for prioritization without any node pooling. This introduces noise into the results and reduces the accuracy of the final ranking, as shown in Section 4 when comparing XGDAG-based variants with standalone XAI tools. In more detail, for any node *n*, the $G_{n}^{LP}$ set is absent in standalone XAI-based prioritization; instead, we use the set $G_{n}^{U}$, which includes genes that are present in the explanation subgraph and that were predicted as unlabeled (U) by the GNN trained in the binary PU setting. Then, we proceed with the scoring and ranking criteria as proposed in Section 3.3. As mentioned earlier, using the entire set of genes predicted as unlabeled for prioritization introduces noise, as it may result in prioritizing genes that are highly unlikely to be associated with the disease, specifically the genes that would be predicted as RN by the GNN trained on the propagated labels. Conversely, the incorporation of label propagation in XGDAG brings additional value by facilitating the learning through pseudo-classes and assisting in the discovery of candidates through LP genes.

## 4 Results

To validate the obtained results, we performed both a numerical evaluation and an enrichment analysis. With the former, we compared, in terms of F1 score, the retrieval effectiveness of XGDAG with other methodologies for gene discovery; we compute the F1 score taking into consideration the number of associated genes in the set of *all associations* that each method is able to detect. Seed genes present in the curated set are not considered for this purpose—they were used as positive genes for the training. This validation setting allows us to test whether our model is able to retrieve genes that had been discovered by previous research. In enrichment analysis, we inspected whether the set of genes prioritized by XGDAG was connected with the diseases under examination, namely whether the genes were enriched in pathways, gene ontologies, or other diseases associated with the considered ones.

### 4.1 Numerical evaluation

**Figure 3.**
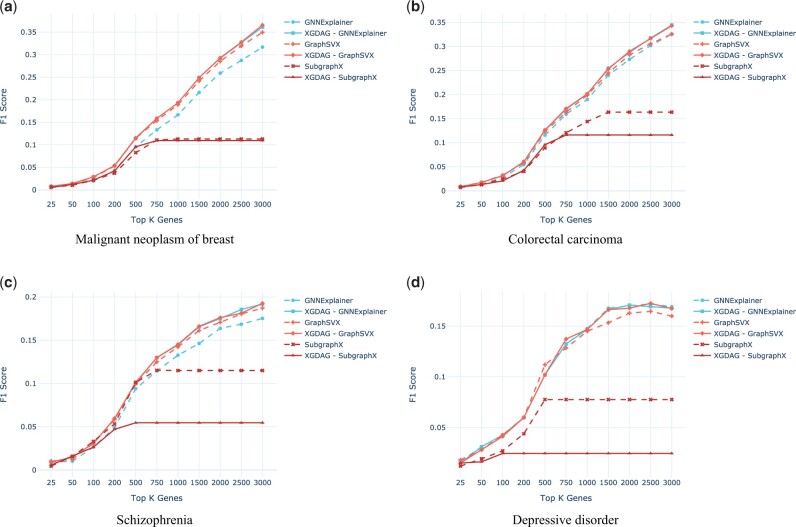
F1 score (*y*-axis) comparison for selected diseases (the remaining ones can be found in the Supplementary Material). The metrics are reported at increasing numbers of retrieved genes (*x*-axis). Dashed lines indicate the standalone XAI method and solid lines the XGDAG version. We notice that using explainability techniques on top of a PU learning prioritization strategy improves significantly the retrieval accuracy of the methods.

First, in Fig. 3, we compare the performance of XGDAG against the single XAI methods on which it is based, used as standalone tools (here we show the F1 score—more comparison metrics are available in the Supplementary Material). Notice that the PU learning-based XAI approach achieves higher performances with respect to its plain-explainability counterpart. Indeed, the use of the pre-prioritization, obtained with the LP set from the label propagation phase, helps in the identification of the pool of possible new candidate genes.

**Figure 4.**
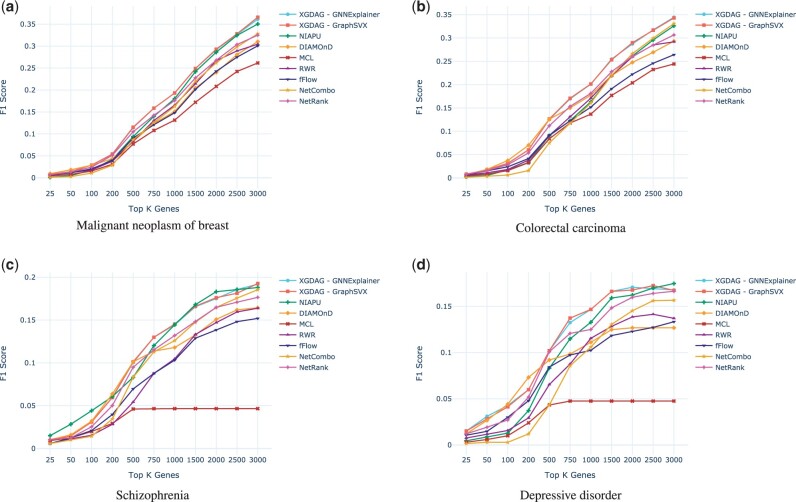
F1 score comparison for selected diseases for the two best-performing XGDAG variants (GNNExplainer and GraphSVX) with known gene discovery methodologies. We notice that when the number of retrieved genes is small the various approaches perform comparably. However, as the number of genes increases, XGDAG remains the most stable and robust method, whereas most of the compared strategies tend to become less accurate in the retrieval. More diseases can be found in the Supplementary Material, together with additional visualizations.

We thus selected the best performing XGDAG variants in terms of overall F1 score. Given their at-par performance, we chose the GraphSVX- and the GNNExplainer-based approaches. We compared them against state-of-the-art methodologies for gene prioritization, namely NIAPU, DIAMOnD, MCL, RWR, two variants of GUILD (fFlow and NetCombo), and ToppGene. The plots in Fig. 4 show that XGDAG is more effective and robust than the other strategies. As we increase the number of retrieved genes, it is able to keep high the number of associated genes retrieved. On the contrary, methodologies such as DIAMOnD may be more effective in the retrieval when a small number of candidates are searched. However, they lose their reliability when higher numbers of candidate genes are considered, as also pointed out by DIAMOnD’s designers (Ghiassian *et al.* 2015). In this, XGDAG proved to be the best solution even when looking for larger sets of candidate genes.

#### 4.1.1 Results on a high-quality curated dataset

By inspecting the results, we noticed the very high accuracy of DIAMOnD on small sets of candidate genes. The dataset we used, even in its curated version, contains a relatively high number of associated genes, some of them not present in other manually curated datasets. We were interested in exploring whether training on datasets with a higher level of curation and smaller numbers of associated genes would change these results.

We performed this additional experiment using the highly curated dataset by Ghiassian *et al.* (2015). This is the dataset on which DIAMOnD was trained and evaluated in the original publication. The PPI network used here was built considering physical interactions validated experimentally and gathered from different sources, as by Menche *et al.* (2015). The GDAs were retrieved from OMIM (Online Mendelian Inheritance in Man) (Hamosh *et al.* 2005) and Genome-Wide Association Studies (GWAS) from PheGenI (Ramos *et al.* 2014). Because of the high-quality level of curation of these GDAs and PPI network, they were used in several gene prioritization experiments (Petti *et al.* 2021, De Luca *et al.* 2022, Gentili *et al.* 2022).

**Figure 5.**
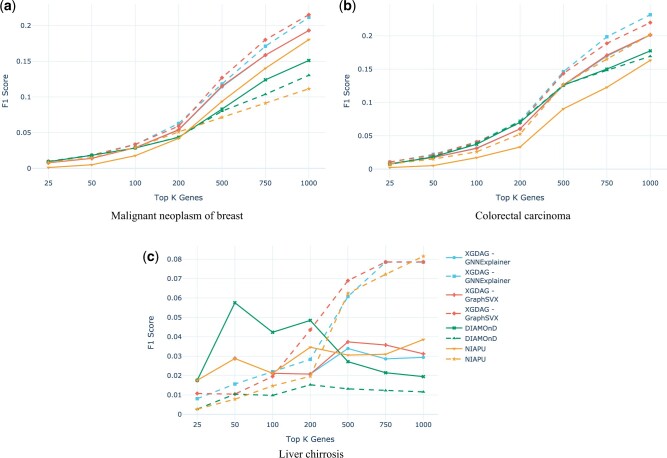
F1 score comparison for the OMIM+PheGenI dataset (dashed line) and the DisGeNET dataset (solid line). Even for a small number of genes, in this experiment XGDAG is competitive against DIAMOnD. The performances on the OMIM+PheGenI dataset are far superior than the DisGeNET ones.

We used the PPI and the GDAs of the aforementioned dataset, which we call *OMIM+PheGenI dataset*, to train the algorithms. We then validated the models on the GDAs from the *all associations* DisGeNET dataset. The goal was to first train the algorithms on high-quality and unbiased data and then test them on an external dataset. For this task, we considered the diseases in common between the two datasets: malignant neoplasm of breast (C0006142), colorectal carcinoma (C0009402), and liver chirrosis (C0023893). A comparative analysis of the F1 score is shown in Fig. 5—additional metrics can be found in the Supplementary Material.

The inspection of the results indicates that training on smaller but better curated datasets is beneficial for XGDAG, whereas DIAMOnD suffers from training on smaller sets of seed genes. This further highlights the robustness of XGDAG whose results are accurate even when the number of seed genes is small. However, the different results obtained when using different datasets demonstrate that data quality plays a major role in gene discovery and prioritization tasks and that a particular focus should be put on the definition of high-quality GDAs and less biased interaction networks (Lazareva *et al.* 2021).

### 4.2 Enrichment analysis

**Figure 6.**
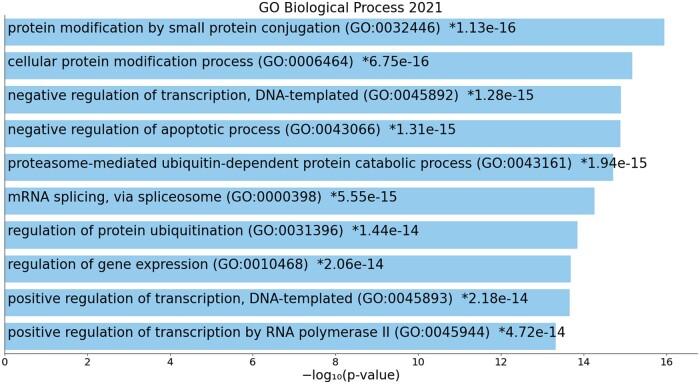
Top 10 significant gene ontologies for disease C0006142 (malignant neoplasm of breast) in the GO Biological Process 2021 database found with Enrichr. Breast cancer-related GOs were retrieved, further proving the effectiveness of XGDAG. Each item is reported with its *p*-value.

As a further analysis to enhance the validity of our methodology, we checked whether the candidate genes retrieved from XGDAG were enriched in biological pathways, gene ontologies (GOs) (Ashburner *et al.* 2000), or other diseases related to the diseases of interest. We provide this analysis for the genes of the DisGeNET dataset prioritized by XGDAG-GNNExplainer. We considered the top 200 genes in our ranking as a reasonable cutoff. We performed the analysis using the Enrichr (Chen *et al.* 2013, Kuleshov *et al.* 2016, Xie *et al.* 2021) web tool and selecting the most statistically significant results according to Fisher’s exact test. For disease C0006142 (malignant neoplasm of breast), several significant gene ontologies and pathways were found. Figure 6 shows the 10 most significant GOs for the biological process domain. Indeed, among the most significant GOs retrieved, protein modification was found to be a potential biomarker in breast cancer (Jin and Zangar 2009). Moreover, dysregulated programs in DNA transcription are related to certain behaviors in cancer cells (Bradner *et al.* 2017). Furthermore, apoptotic process regulation plays an important role in cancer progression and therapies (Reed 2003, Plati *et al.* 2011, Pfeffer and Singh 2018). Enrichment analysis proved genes retrieved by XGDAG to have meaningful associations to the disease. Summarized results for the 10 studied diseases providing the most enriched pathway, ontology, or associated disease and reference papers confirming the findings can be found in the Supplementary Material.

## 5 Discussion and conclusions

In this work, we propose a new methodology, XGDAG, which relies on PU learning, GNNs, and explainability to detect novel GDAs by providing a prioritization of candidates. XGDAG uses a set of effective features defined in previous work (Stolfi *et al.* 2023) to enable PU learning by assigning pseudo-classes to unlabeled instances. This information is then leveraged by our GNN, which is able to generate network topology-aware embeddings that allow for high accuracy predictions. In this context, accurate but black-box models do not provide any additional information than what we already know about gene associations. Thus, given that the reliability of the explanations will depend on the quality of the model itself, an accurate model is the base from which we start our explanation phase. The application of several XAI techniques (among which GNNExplainer and GraphSVX are the most effective) opens the black box on the GNN by determining the most influential nodes for the prediction. Some of these nodes are present in the set of genes predicted as LP: these nodes are selected as new candidate genes.

This is a novel use of XAI. Generally, the main goal of explainability is to gain insights into the decision process of a model. Diversely, in our approach, we exploit XAI methods to draw the final ranking of candidate genes, with the added value of having an interpretable output. This is a novelty that presents XAI not only as a tool that opens the black box of deep neural networks but also as an analysis component directly incorporated into the GDA discovery pipeline tasked with producing the final output.

The method outperforms state-of-the-art methodologies for gene discovery demonstrating the effective synergy of PU learning and explainability on GNN models. The XGDAG results are stable and robust, even considering large numbers of candidate genes.

It is interesting to point out that by using datasets with an in-depth level of manual curation, such as the one by Ghiassian *et al.* (2015), the retrieval performance of XGDAG increases, demonstrating both the robustness of the approach and the importance of curated data.

Additionally, enrichment analysis uncovers associated pathways, ontologies, and traits linked to the selected diseases, backing up the accuracy of the gene ranking obtained with XGDAG and further proving its effectiveness as a gene discovery strategy.

Our approach is based on the analysis of general graph-structured data, so it can be applied in various settings based on network modeling. Future directions can concentrate on the application of XGDAG on multiplex networks (Halu *et al.* 2019) and multi-omics data (Krassowski *et al.* 2020). Notably, datasets such as the Omics Discovery Index (Perez-Riverol *et al.* 2017, 2019), and ConsensusPathDB (Kamburov *et al.* 2009, 2013, Kamburov and Herwig 2022) combine information from proteomics, metabolomics, genomics, and other interaction networks; expanding the study to encompass this type of data can further enhance the insights acquired through our methodology.

Finally, our study suggests that efforts can be put into the development of PU learning and XAI techniques devoted to GNNs for gene discovery purposes, giving the rewarding results that can be obtained by the joint use of such methods. The main limitation, as we observed in Section 4.1, is the requirement of high-quality data (Lazareva *et al.* 2021). This is of course shared by all data-based computational approaches; however, as more genes are discovered and validated, the results will be more trustworthy.

## Supplementary Material

btad482_Supplementary_Data
