# Supplementary material for: XGDAG: explainable gene–disease associations via graph neural networks
Source: Bioinformatics. 2023 Aug 2;39(8):btad482. doi: 10.1093/bioinformatics/btad482 (PMC10421968; doi:10.1093/bioinformatics/btad482)
Supplement: btad482_Supplementary_Data [file btad482_supplementary_data.pdf]

Supplementary Material to "XGDAG: eXplainable Gene–Disease Associations via Graph Neural Networks"

Andrea Mastropietro[1], Gianluca De Carlo[1] and Aris Anagnostopoulos[1]

[1]Department of Computer, Control and Management Engineering "Antonio Ruberti", Sapienza University of Rome, Rome 00185, Italy

1 Structural analysis of network properties

This supplementary section provides an analysis of the structural properties of the BioGRID PPI network used, along with the disease modules, enriched with the first neighbors for deeper analysis. In Table 1 we report the following structural properties (starting from the *nodes* column, the values are related to the largest connected component): seeds (number of known associated genes), CC (number of connected components), number of nodes and edges, average degree, radius, diameter, density, average shortest path length, clustering coefficient, average betweenness, closeness, and eigenvector centrality measures.

Table 1: Analysis of structural properties of the BioGRID PPI and the disease modules enriched with the set of first neighbors.

	Seeds	CC	Nodes	Edges	Avg. degree	Rad.	Diam.	Dens.	Avg. Short. path	Clust. coeff.	Avg. betwenness centrality	Avg. closeness centrality	Avg. eigenvector centrality
PPI	N/A	4	19,761	67,8932	68.714	4	7	0.003	2.804	0.115	9.132e-5	0.361	0.003
Disease modules enriched with first neighbors													
C0860207	320	1	9,907	34,772	7.02	4	7	0.0007	3.483	0.117	0.0003	0.290	0.005
C0005586	451	2	12,160	42,841	7.046	4	8	0.0006	3.332	0.127	0.0002	0.304	0.004
C3714756	431	2	11,079	46,761	8.441	4	7	0.0008	3.488	0.111	0.0002	0.290	0.004
C0001973	255	3	8,889	20,609	4.637	4	8	0.0005	3.453	0.147	0.0003	0.294	0.005
C0376358	606	1	14,187	85,308	12.026	4	7	0.0008	0.180	3.118	0.0001	0.325	0.004
C0011581	279	2	10,625	27,993	5.269	4	8	0.0005	3.424	0.092	0.0002	0.296	0.005
C0023893	747	2	13,327	63,366	9.509	4	8	0.0007	3.386	0.093	0.0002	0.299	0.004
C0006142	1025	4	15,012	115,606	15.402	4	7	0.001	3.146	0.152	0.0001	0.322	0.004
C0009402	672	2	13,373	65,482	9.793	4	7	0.0007	3.277	0.131	0.0002	0.310	0.004
C0036341	832	2	13,654	75,909	11.119	4	8	0.0008	3.294	0.137	0.0002	0.308	0.004

2 Further details on NIAPU

NIAPU (Stolfi et al. (2023)) makes use of the NeDBIT (Network Diffusion and Biology-Informed Topological) features. Those features are comprised of two network diffusion-based features (heat diffusion and balanced diffusion) and two biology-informed topological features (NetShort and NetRing). Proper references are given in the main paper. Differently from classic network measures, NeDBIT features are built taking into account the seed genes of a disease, being thus more relevant and descriptive for the disease considered. The first feature is obtained via a heat diffusion process over the network. Starting with a distribution of weights with positive values on seed genes, their evolution is determined by using a diffusion equation on graph. Balanced diffusion is a similar feature. The difference with the previous one is that the same amount of score diffuses for each node, while for heat diffusion the same amount of score is diffused for each edge. The NetShort measure relies on the idea that a node is topologically important for a disease if a large number of seed nodes must be traversed to reach it. Finally, NetRing is based on the concept of rings. Starting from seed nodes, a partition of the graph in sub-graphs, or rings is defined. This measure rewards non-seed nodes

that are close to high-ranking nodes in the previous ring and that are linked with a few nodes of the same or higher ring. A node has a high rank if it has many seed nodes as neighbors.

The NIAPU pipeline is composed of six main steps and uses a Markovian diffusion process to assign pseudo-labels to unlabeled elements. The steps were already introduced in the main paper, but we give more details in this supplementary section, as also reported in the original NIAPU paper.

We consider V as the set whose i^{th} element $v_{i=1,\dots,n}$ is characterized by $(\boldsymbol{x}_i, y_i)$ where $\boldsymbol{x}_i$ is the feature vector describing the gene, and y_i the initial (binary) label (positive or unlabeled). The first step deals with the definition of the similarity matrix between the genes. The symmetric matrix $\boldsymbol{W}$ is such as the element w_{ij} is defined as

$$w_{ij} = \begin{cases} 1 - \frac{e_{ij}-m}{M-m} & \text{if } i \neq j \\ 1 & \text{otherwise} \end{cases} \tag{1}$$

where $e_{ij} = \sum_k \left(x_i^k - x_j^k\right)^2$, $m = \min_{ij}\{e_{ij}\}$ and $M = \max_{ij}\{e_{ij}\}$. The symmetric matrix $\boldsymbol{W}$ contains the similarity score between elements i and j. In the second step, the matrix is reduced as

$$w_{r,ij} = \begin{cases} w_{ij} & \text{if } w_{ij} > q_{\boldsymbol{w}} \\ 0 & \text{otherwise} \end{cases}, \tag{2}$$

where $q_{\boldsymbol{w}}$ is a threshold value computed as a quantile of the distribution of the elements in the matrix $\boldsymbol{W}$ to exclude from the propagation process edges with weak connections. The matrix $\boldsymbol{W}_r$ is then normalized as $\boldsymbol{W}_n = \boldsymbol{D}^{-1}\boldsymbol{W}_r$, where $\boldsymbol{D}$ is the diagonal matrix with elements $d_{ii} = \sum_j w_{r,ij}$.

In the third step, the propagation process is initialized with the initial state vector $\boldsymbol{g}_0$. Let $|P|$ be the cardinality of the set of positive genes P and $\hat{\boldsymbol{x}} = \left(\hat{x}^1, \dots, \hat{x}^d\right)$, where $\hat{x}^k = 1/|P| \sum_{i \in P} x_i^k$, be the average features of P. The RN (reliable negative) genes are chosen as the ones having the most distant features from $\hat{\boldsymbol{x}}$. If $|P|$ most distant genes from $\hat{\boldsymbol{x}}$ are chosen, then the i-th element of $\boldsymbol{g}_0$ is defined as

$$g_{0,i} = \begin{cases} 1 & \text{if} \quad i \in P \\ -1 & \text{if} \quad i \in RN \\ 0 & \text{otherwise} \end{cases}. \tag{3}$$

If a different number of RN genes are selected, their initial values must be set such that the sum of the elements in $\boldsymbol{g}_0$ is 0. In our work, $|RN|$ is set to contain 20% of the genes.

The fourth step of the pipeline is the definition of the Markov process:

$$\boldsymbol{g}_r = (1-\alpha)\,\boldsymbol{W_n^t}\boldsymbol{g}_{r-1} + \alpha\boldsymbol{g}_0. \tag{4}$$

The parameter α is set to 0.8 in the original publication. The diffusion process starts from $\boldsymbol{g}_0$ and converges to the stationary distribution $\boldsymbol{g}_\infty$.

In the fifth step, G_∞ is used to assign the remaining pseudo-labels LP, WN, and LN. The values G_∞ are ranked (excluding P and RN elements). The criterion applied (proposed in the original paper and used in our application) is to split the ranking into three equal parts and identify LP samples with the first third, WN with the second third, and LN with the last third (for class balancing).

The last step is the classification using an ML model trained over the newly assigned labels; our pipeline makes use of a GNN, as described.

3 Model selection

Since the GNN is the base that will be used by the XAI methods, we needed to obtain a model that was as accurate as possible in the detection of the positive (P) and likely positive (LP) samples. We thus performed a model selection via a competitive study on the performances on the validation set (15% of the whole dataset) in order to select the best model to use in our pipeline. We inspected different GNN architectures, numbers of layers (depth), and aggregation functions. Figure 1 shows the average performances of the different models inspected.

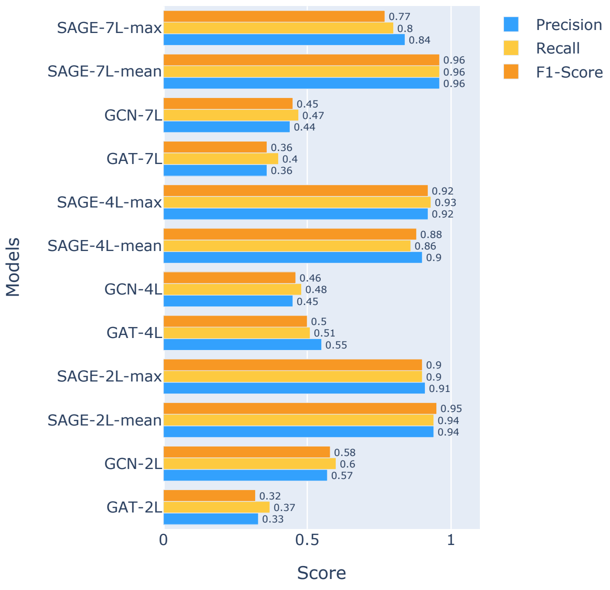

Figure 1: Performance comparison of different architectures. The best-performing model was the 7-layer GraphSAGE-based GNN with the mean aggregator.

Moreover, Figure 2 shows the training times of each GNN model. Even though being slightly slower than shallower architectures, the 7-layer GraphSAGE model (with mean aggregator) was chosen as the final model considering the positive trade-off between training time and model accuracy. Notably, the training time reported (around 30 minutes) is more than acceptable for a GNN. Moreover, given that the task of the method is to discover new GDAs in a non-time-sensitive scenario, we aim for a very accurate model that can potentially find new genes even at the cost of a slightly longer training time. The training was executed on a machine with an NVIDIA RTX 3070 GPU with 8 GB of dedicated memory, an AMD Ryzen 7 5800H CPU (up to 4.4GHz), and 16 GB of DDR4 RAM.

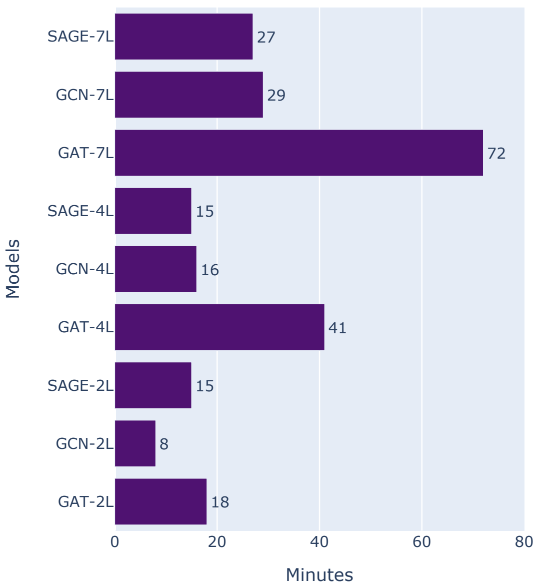

Figure 2: Training times of the different GNN models. Given that the differences in the training times of the max and mean aggregator versions of the GraphSAGE models are negligible, we report them only once.

3.1 Scalability of the XAI methods

As shown in Figure 3, we preformed a scalability analysis of the different XGDAG variants (the standalone methods scale analogously). The average explanation times for a single node are 35.54 seconds for XGDAG-GNNExplainer, **24.36 seconds** for XGDAG-GraphSVX, 49.34 seconds for XGDAG-SubgraphX. It has to be noted that, when using SubgraphX, due to its long computation time, the explained nodes were limited only to such nodes with at most 20 neighbors. Even by limiting the size of the neighborhoods, SubgraphX takes twice the time needed by GraphSVX, proving unfeasible for explaining nodes in dense networks.

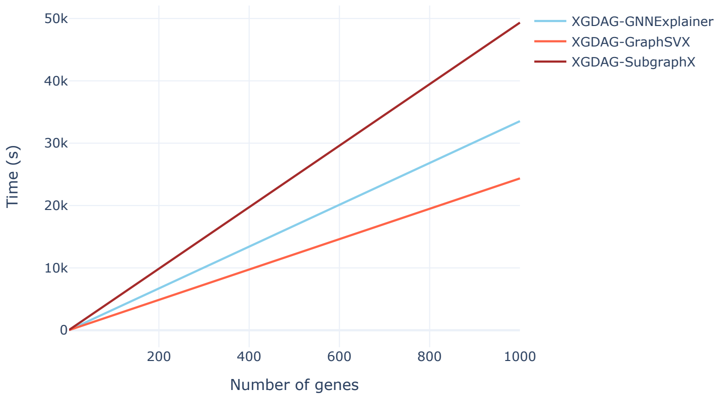

Figure 3: Average explaination time. The time increase linearly with respect to the number of genes/nodes explained.

4 Additional results

In this supplementary section, we present the results obtained on the diseases not shown in the main paper (Figure 4), along with additional metrics. More in detail, Figures 5a and 5b show the precision comparison between the standalone XAI methods and XGDAG. Figures 6a and 6b show the same comparison for the recall metric. We notice how for some diseases the scores are low for all the methodologies; for those cases, it is hard to make a clear comparison. This trend also happens for other state-of-the-art computational methods, as one can see from the heatmaps in Figures 7a and 7b and the line plots in Figure 8. As aforementioned, there are some challenging diseases for which all the strategies perform poorly. This mainly happens when the number of known associations is low; this impinges on the generalization power of the methods and points to the need for high-quality data. Finally, Figures 9 and 10 show respectively precision and recall scores for the comparison between DisGeNET and OMIM+PheGenI datasets.

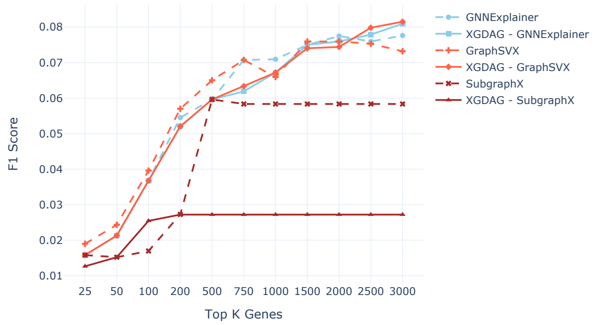

(a) Bipolar disorder

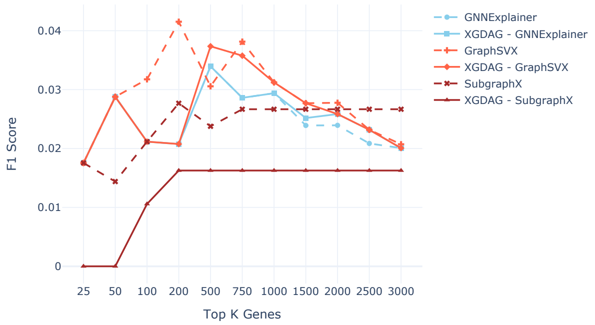

(b) Liver cirrhosis

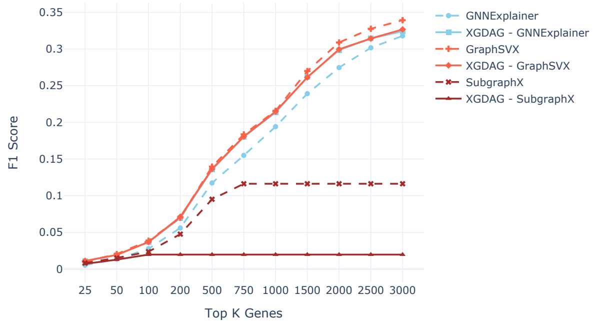

(c) Malignant neoplasm of prostate

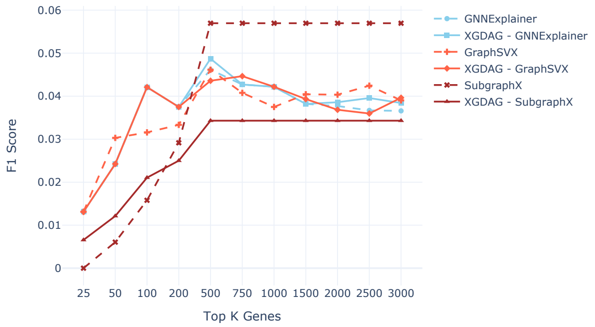

(d) Chronic alcoholic intoxication

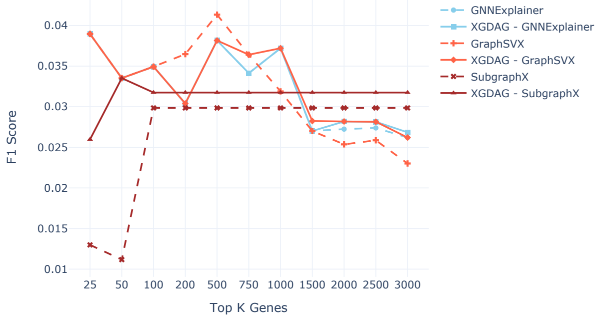

(e) Drug-induced liver disease

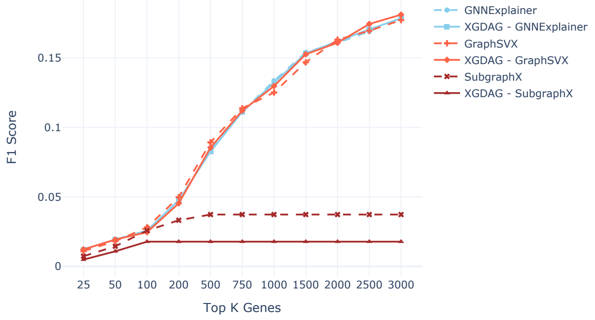

(f) Intellectual disability

Figure 4: F1 score (y-axis) comparison for the disease not present in the main paper. The metrics are reported at increasing numbers of retrieved genes (x-axis). Dashed lines indicate the standalone XAI method and solid lines the XGDAG version.

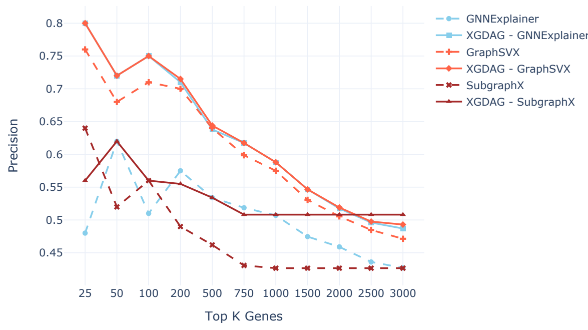

(a) Malignant neoplasm of breast

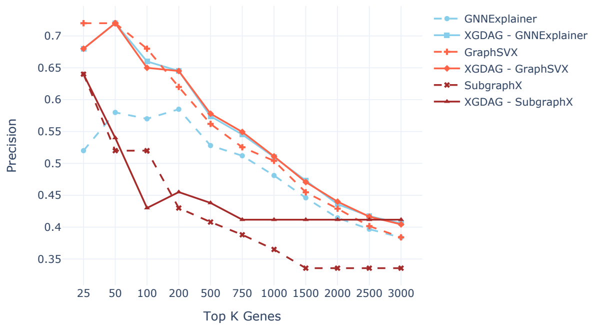

(b) Colorectal carcinoma

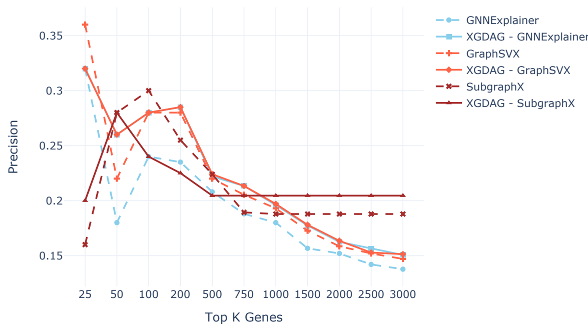

(c) Schizophrenia

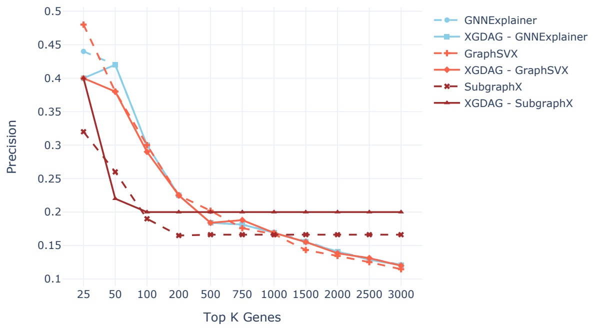

(d) Depressive disorder

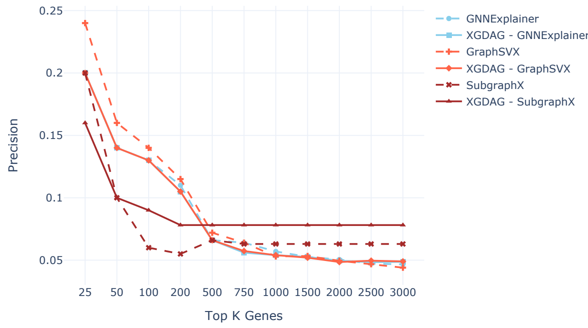

(e) Bipolar disorder

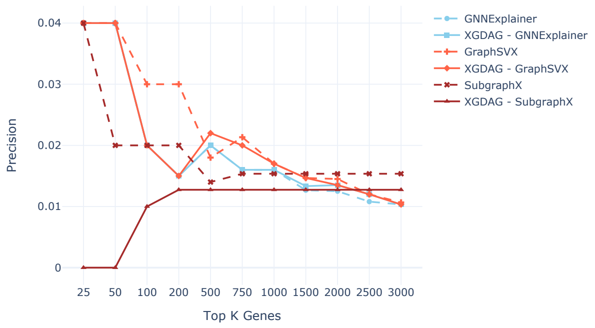

(f) Liver cirrhosis

Figure 5a: Precision (y-axis) comparison. The metrics are reported at increasing numbers of retrieved genes (x-axis). Dashed lines indicate the standalone XAI method and solid lines the XGDAG version.

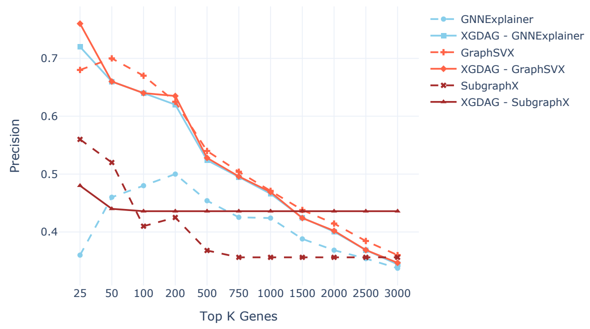

(a) Malignant neoplasm of prostate

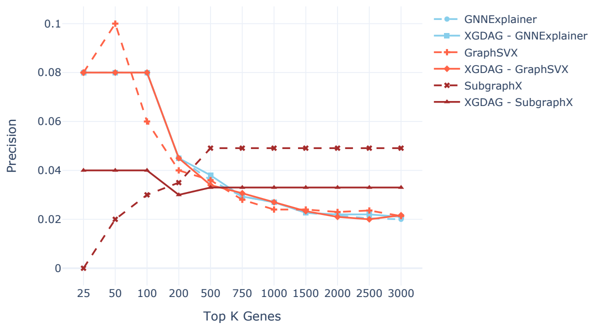

(b) Chronic alcoholic intoxication

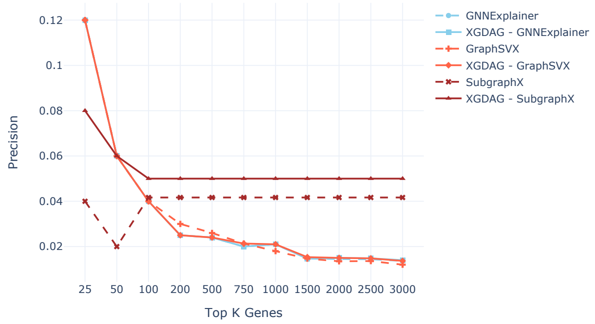

(c) Drug-induced liver disease

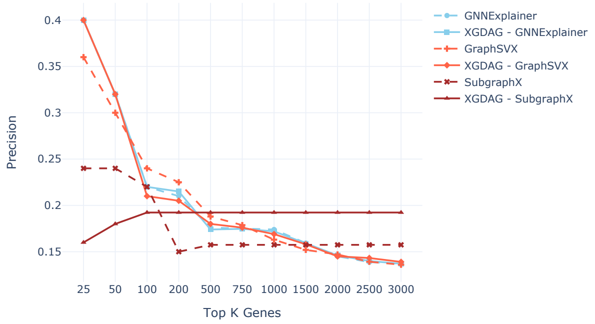

(d) Intellectual disability

Figure 5b: Precision (y-axis) comparison. The metrics are reported at increasing numbers of retrieved genes (x-axis). Dashed lines indicate the standalone XAI method and solid lines the XGDAG version.

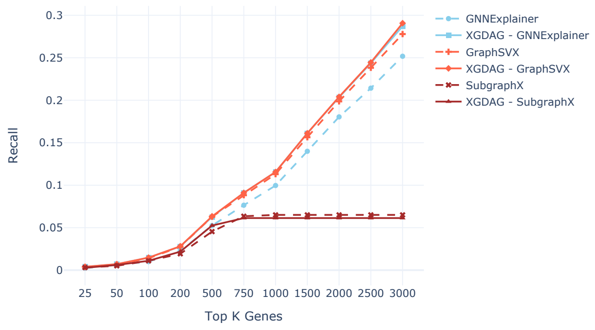

(a) Malignant neoplasm of breast

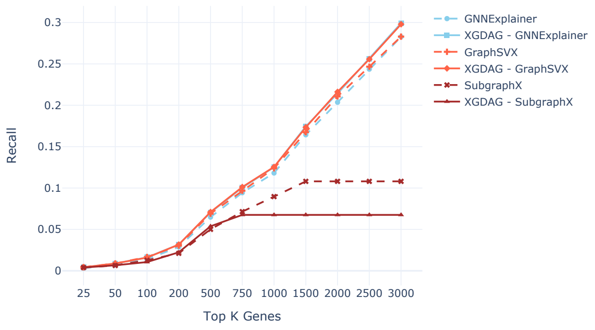

(b) Colorectal carcinoma

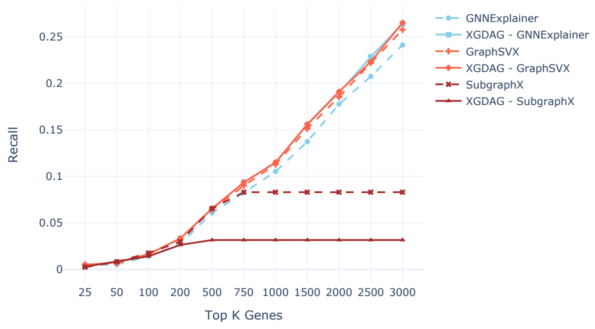

(c) Schizophrenia

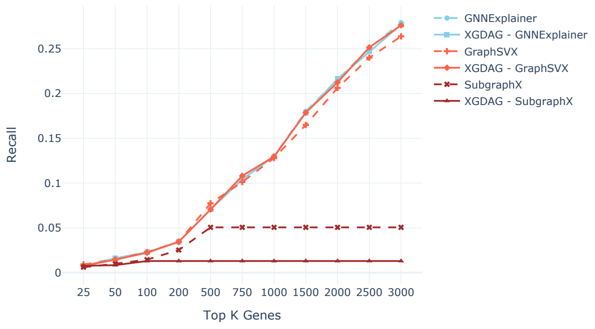

(d) Depressive disorder

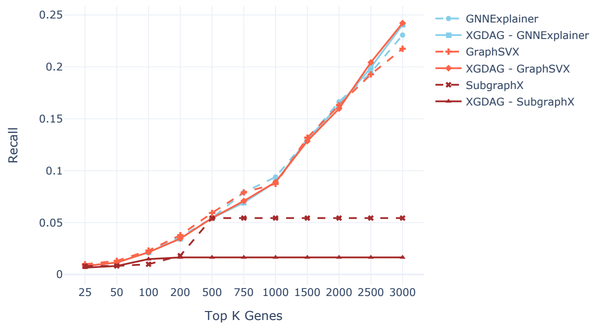

(e) Bipolar disorder

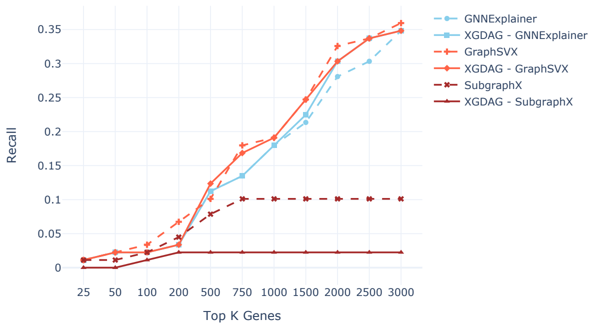

(f) Liver cirrhosis

Figure 6a: Recall (y-axis) comparison. The metrics are reported at increasing numbers of retrieved genes (x-axis). Dashed lines indicate the standalone XAI method and solid lines the XGDAG version.

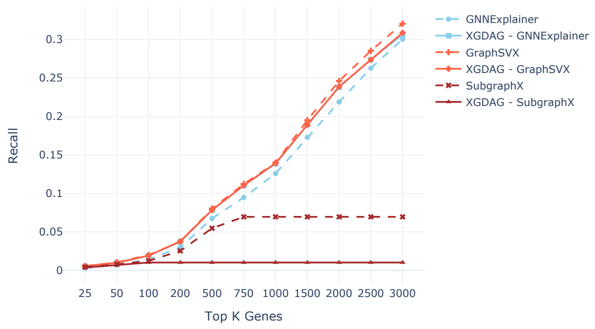

(a) Malignant neoplasm of prostate

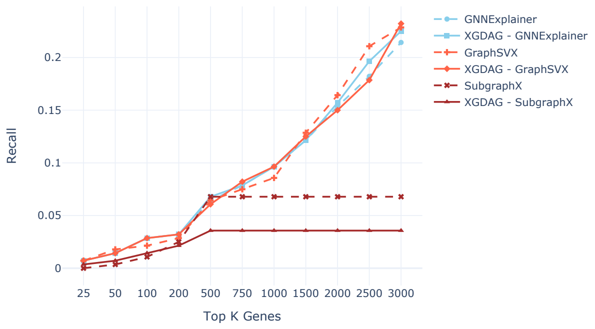

(b) Chronic alcoholic intoxication

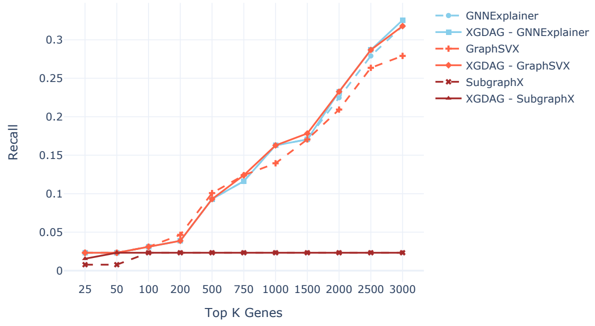

(c) Drug-induced liver disease

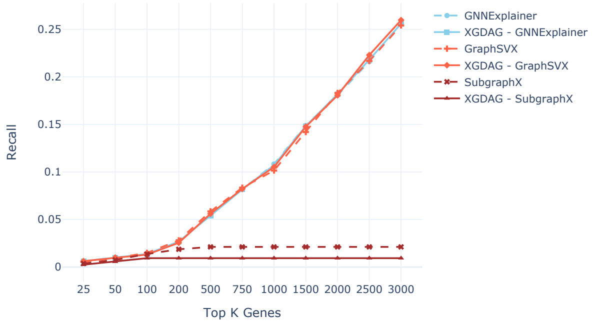

(d) Intellectual disability

Figure 6b: Recall (y-axis) comparison. The metrics are reported at increasing numbers of retrieved genes (x-axis). Dashed lines indicate the standalone XAI method and solid lines the XGDAG version.

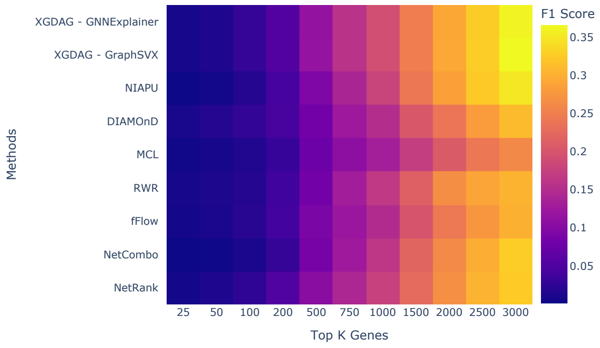

(a) Malignant neoplasm of breast

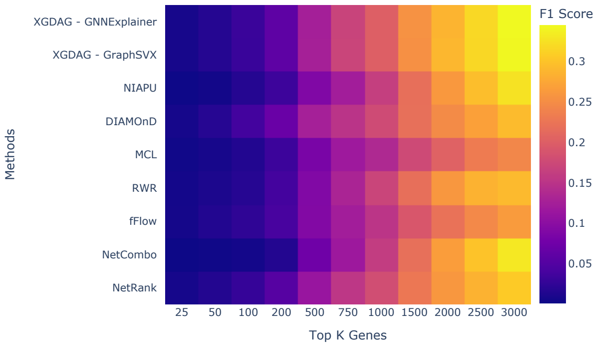

(b) Colorectal Carcinoma

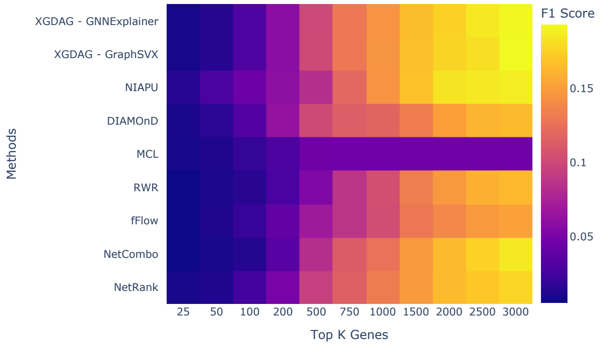

(c) Schizophrenia

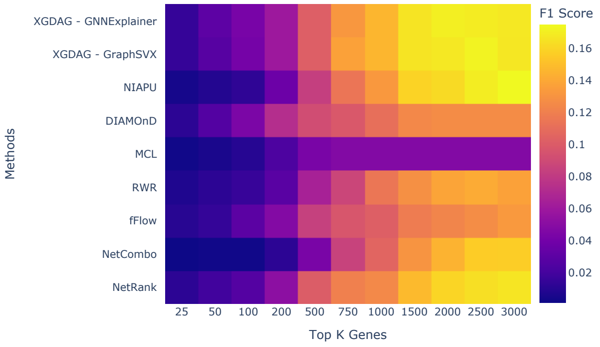

(d) Depressive disorder

Figure 7a: F1 score comparison (heatmaps) for the diseases present in the main paper for the two best-performing XGDAG variants (GNNExplainer and GraphSVX) with known gene discovery methodologies.

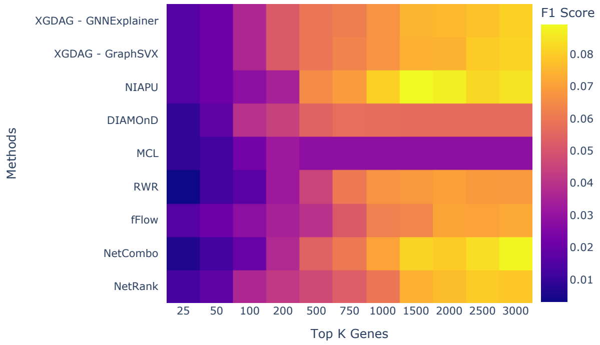

(a) Bipolar disorder

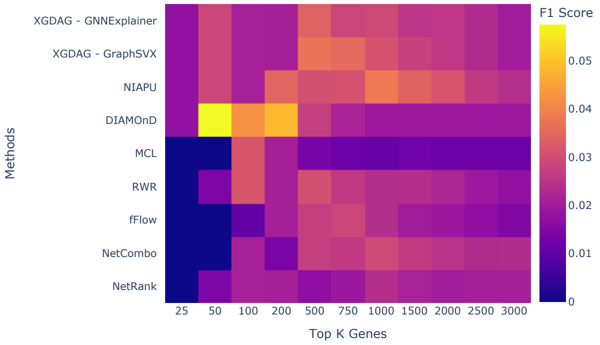

(b) Liver cirrhosis

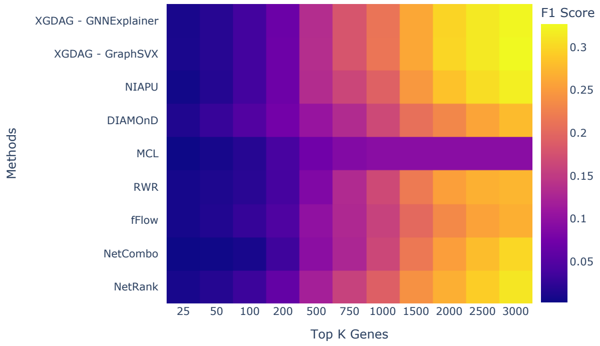

(c) Malignant neoplasm of prostate

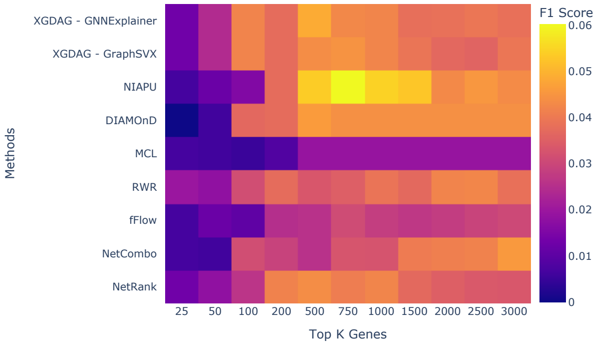

(d) Chronic alcoholic intoxication

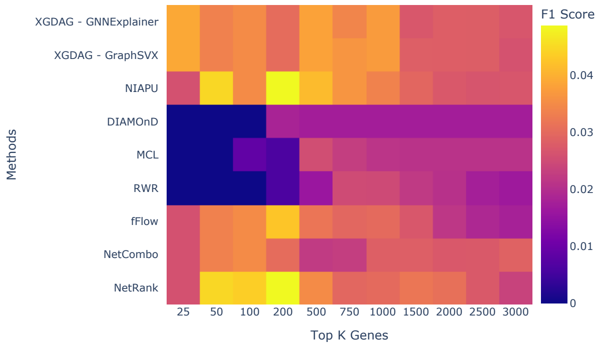

(e) Drug-induced liver disease

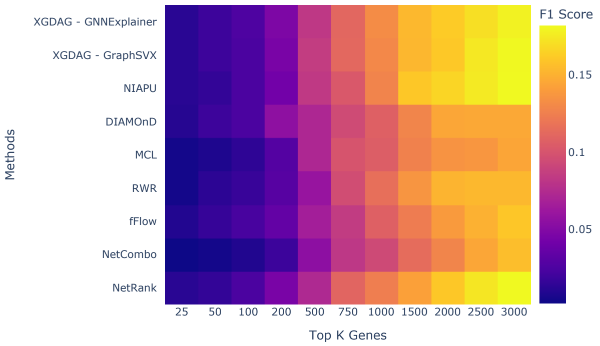

(f) Intellectual disability

Figure 7b: F1 score comparison (heatmaps) for the disease not present in the main paper for the two best-performing XGDAG variants (GNNExplainer and GraphSVX) with known gene discovery methodologies.

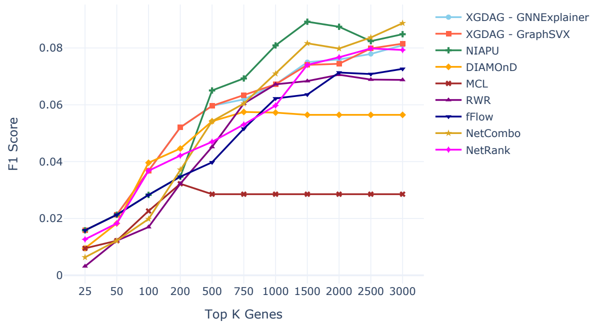

(a) Bipolar disorder

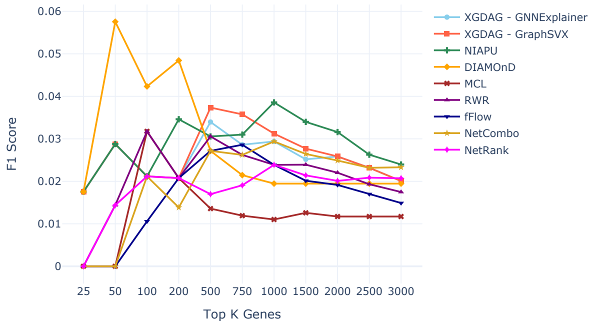

(b) Liver cirrhosis

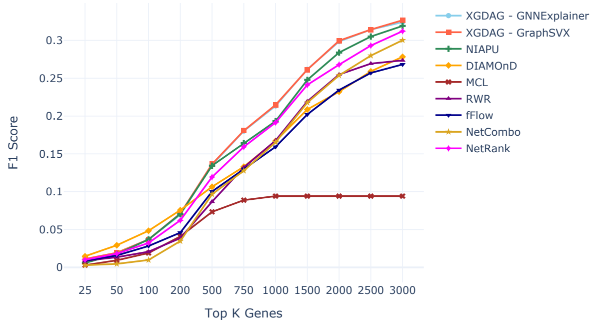

(c) Malignant neoplasm of prostate

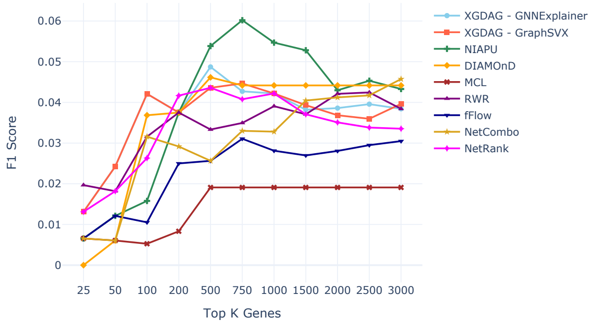

(d) Chronic alcoholic intoxication

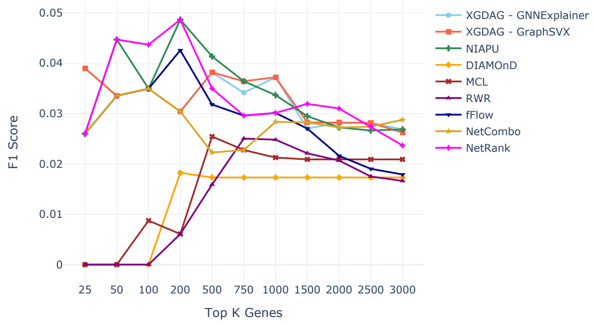

(e) Drug-induced liver disease

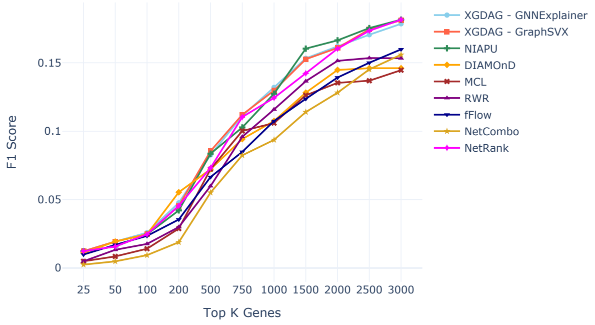

(f) Intellectual disability

Figure 8: F1 score comparison (line plots) for the disease not present in the main paper diseases for the two best-performing XGDAG variants (GNNExplainer and GraphSVX) with known gene discovery methodologies.

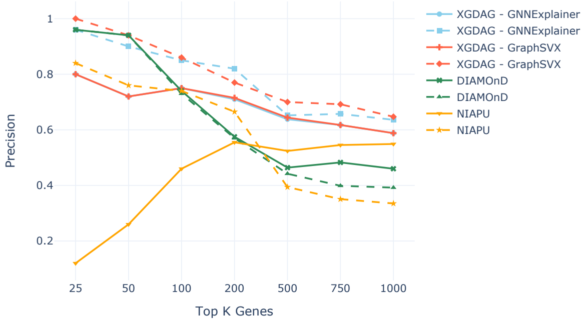

(a) Malignant neoplasm of breast

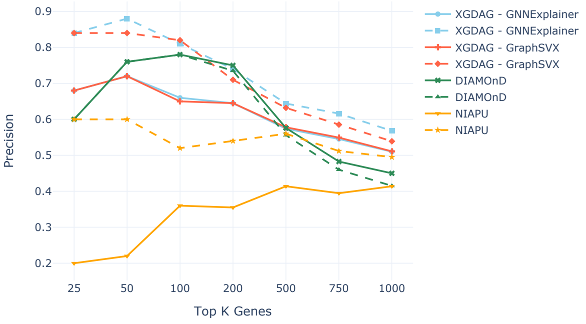

(b) Colorectal carcinoma

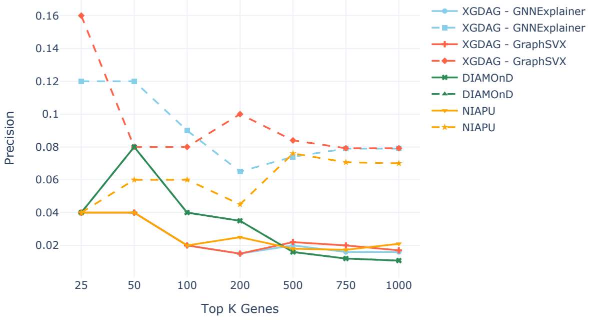

(c) Liver chirrosis

Figure 9: Precision comparison for the OMIM+PheGenI dataset (dashed line) and the DisGeNET dataset (solid line).

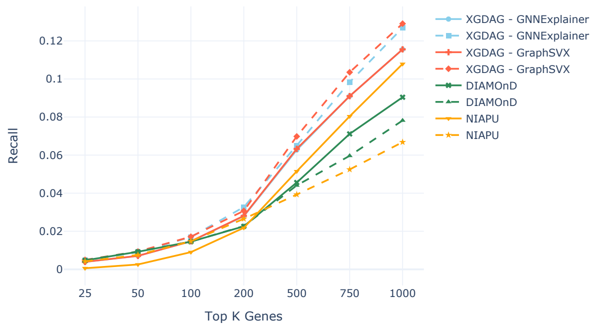

(a) Malignant neoplasm of breast

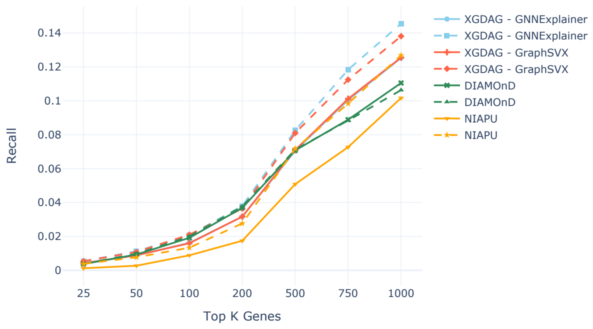

(b) Colorectal carcinoma

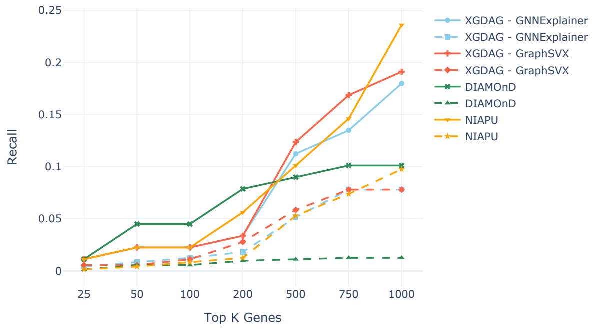

(c) Liver chirrosis

Figure 10: Recall comparison for the OMIM+PheGenI dataset (dashed line) and the DisGeNET dataset (solid line).

5 Enrichment analysis

We hereby report in Table 2 the enrichment analysis results for the ten diseases studied, showing the most significant pathway, ontology, or related disease.

Table 2: Enrichment analysis for the considered diseases. We report the most enriched GO, pathway, or disease.

Disease	Top enriched term (GO/disease/pathway)	Details	References
C0006142<br>Malignant neoplasm of breast	GO:0032446<br>Protein modification by small protein conjugation	Protein modification was found to be a biomarker in breast cancer.	Jin & Zangar (2009)
C0036341<br>Schizophrenia	Androgen receptor signaling pathway	Altered androgen receptor activity may impact stress in men with schizophrenia.	Owens et al. (2019)
C0023893<br>Liver cirrhosis	GO:0042981<br>Regulation of apoptotic process	Apoptosis is a typical pathological feature of liver diseases and excessive apoptosis can generate acute liver injuries.	Wang (2014)<br>Guicciardi & Gores (2005)
C0009402<br>Colorectal carcinoma	GO:0006464<br>Cellular protein modification process	Protein synthesis deregulation is a frequent event in cancer, and many colorectal cancer mutations are responsible for the deregulation of translational processes.	Schmidt et al. (2020)
C0376358<br>Malignant neoplasm of prostate	GO:0043066<br>Negative regulation of apoptotic process	The ability of cells to avoid apoptosis is crucial in cancer development and anti-apoptotic pathways play a major role in the development of effective treatments.	McKenzie & Kyprianou (2006)<br>Ali & Kulik (2021)
C0005586<br>Bipolar disorder	Amyotrophic lateral sclerosis (ALS)	Hospitalized patients with bipolar disorder and psychiatric conditions were significantly associated with a first ALS diagnosis within a year.	Turner et al. (2016)
C3714756<br>Intellectual disability	Neurodevelopmental disorder (Au-Kline Syndrome)	Au-Kline syndrome affects different body systems leading to intellectual disability, hypotonia, and delayed development.	Au et al. (2018)
C0860207<br>Drug-induced liver disease	Messenger RNA processing	A consistent number of circulating Messenger RNA and other microRNAs in plasma collected from drug-overdosed animals are found to be highly expressed in the liver.	Wang et al. (2009)
C0011581<br>Depressive disorder	GO:0043066<br>Negative regulation of apoptotic process	Major depressive disorder shows evidence of local inflammatory, apoptotic, and oxidative stress.	Shelton et al. (2011)
C0001973<br>Chronic alcoholic intoxication	Dementia	Chronic abuse of alcohol can cause structural and functional brain damage, leading to alcohol-related dementia.	Sachdeva et al. (2016)

References

Ali, A. & Kulik, G. (2021), 'Signaling pathways that control apoptosis in prostate cancer', *Cancers* **13**(5), 937.

Au, P., Goedhart, C., Ferguson, M., Breckpot, J., Devriendt, K., Wierenga, K., Fanning, E., Grange, D. K., Graham, G. E., Galarreta, C. et al. (2018), 'Phenotypic spectrum of au–kline syndrome: A report of six new cases and review of the literature', *European Journal of Human Genetics* **26**(9), 1272–1281.

Guicciardi, M. & Gores, G. (2005), 'Apoptosis: a mechanism of acute and chronic liver injury', *Gut* **54**(7), 1024–1033.

Jin, H. & Zangar, R. C. (2009), 'Protein modifications as potential biomarkers in breast cancer', *Biomarker insights* **4**, BMI–S2557.

McKenzie, S. & Kyprianou, N. (2006), 'Apoptosis evasion: the role of survival pathways in prostate cancer progression and therapeutic resistance', *Journal of cellular biochemistry* **97**(1), 18–32.

Owens, S. J., Weickert, T. W., Purves-Tyson, T. D., Ji, E., White, C., Galletly, C., Liu, D., O'Donnell, M. & Weickert, C. S. (2019), 'Sex-specific associations of androgen receptor cag trinucleotide repeat length and of raloxifene treatment with testosterone levels and perceived stress in schizophrenia', *Complex Psychiatry* **5**(1), 28–41.

Sachdeva, A., Chandra, M., Choudhary, M., Dayal, P. & Anand, K. S. (2016), 'Alcohol-related dementia and neurocognitive impairment: a review study', *International journal of high risk behaviors & addiction* **5**(3).

Schmidt, S., Denk, S. & Wiegering, A. (2020), 'Targeting protein synthesis in colorectal cancer', *Cancers* **12**(5), 1298.

Shelton, R., Claiborne, J., Sidoryk-Wegrzynowicz, M., Reddy, R., Aschner, M., Lewis, D. & Mirnics, K. (2011), 'Altered expression of genes involved in inflammation and apoptosis in frontal cortex in major depression', *Molecular psychiatry* **16**(7), 751–762.

Stolfi, P., Mastropietro, A., Pasculli, G., Tieri, P. & Vergni, D. (2023), 'NIAPU: Network-Informed Adaptive Positive-Unlabeled learning for disease gene identification', *Bioinformatics* . btac848.
URL: *https://doi.org/10.1093/bioinformatics/btac848*

Turner, M. R., Goldacre, R., Talbot, K. & Goldacre, M. J. (2016), 'Psychiatric disorders prior to amyotrophic lateral sclerosis', *Annals of neurology* **80**(6), 935–938.

Wang, K. (2014), 'Molecular mechanisms of hepatic apoptosis', *Cell death & disease* **5**(1), e996–e996.

Wang, K., Zhang, S., Marzolf, B., Troisch, P., Brightman, A., Hu, Z., Hood, L. E. & Galas, D. J. (2009), 'Circulating micrornas, potential biomarkers for drug-induced liver injury', *Proceedings of the National Academy of Sciences* **106**(11), 4402–4407.
